# Integrative transcriptomic and proteomic profiling reveals altered thymocyte development and microenvironment remodeling during natural thymic atrophy

**DOI:** 10.3389/fcell.2026.1816627

**Published:** 2026-06-25

**Authors:** Wei Lin, Fuju Sun, Haitao Pan, Jiali Yao, Mengyao Wang, Kang Ye, Jing Yang

**Affiliations:** 1 Tongde Hospital of Zhejiang Province Affiliated to Zhejiang Chinese Medical University (College of Integrated Traditional Chinese and Western Medicine Clinical Medicine), Hangzhou, China; 2 Zhejiang Academy of Traditional Chinese Medicine, Hangzhou, China; 3 Pharmacology Research Center, Zhejiang Shouxiangu Botanical Drug Institute, Hangzhou, China; 4 Pharmacology Research Center, Zhejiang Research Institute of Traditional Chinese Medicine, Hangzhou, China

**Keywords:** immunosenescence, proteomics, thymic atrophy, thymocyte development, transcriptomics

## Abstract

**Background:**

Age-related thymic atrophy (ARTA) is a hallmark of immunosenescence, yet the earliest thymocyte developmental checkpoints affected by increasing age and the coordinated molecular programs that drive thymic degeneration remain incompletely defined.

**Methods:**

We compared young (1-month-old) and middle-aged (MA, 12-month-old) male ICR mice using thymus weight/index measurement, histopathology, peripheral blood cell analysis, and immunostaining of thymic markers. We further performed RNA-seq and data-dependent acquisition (DDA) proteomics, followed by integrated transcriptomic-proteomic pathway analyses. Finally, we analyzed public human thymus datasets to assess the translational relevance of our findings.

**Results:**

Middle-aged mice exhibited marked thymic involution with reduced thymus weight and thymic index, accompanied by peripheral lymphopenia and reduced peripheral T-cell counts, while myeloid populations (neutrophils and monocytes) increased. Pathological examination revealed lipid droplet accumulation in the thymus of aged mice, along with decreased Ki-67 expression and an increased number of apoptotic cells. Histologically, aged thymuses showed cortical thinning and an indistinct corticomedullary boundary. Reduced cortical CD25 with increased CD44 is suggestive of a possible developmental impediment around the DN1-to-DN2 transition; in parallel, CD3^+^, CD4^+^, and CD8^+^ T cells were reduced in MA mice. Transcriptomics identified broad remodeling (2,084 upregulated and 255 downregulated genes), featuring heightened inflammatory responses, extracellular matrix (ECM)-receptor interaction, and fatty acid metabolism, with suppression of DNA replication-related programs. Proteomics revealed concordant shifts (189 upregulated and 91 downregulated proteins), including enhanced metabolic and ECM-related pathways and reduced DNA replication and T-cell differentiation signatures. Integrated multi-omics highlighted 289 synchronously upregulated gene-protein pairs enriched in focal adhesion, PI3K/Akt signaling, ECM-receptor interaction, and complement/coagulation cascades, indicating coordinated microenvironmental injury and remodeling during thymic atrophy. In the translational relevance analysis, the aging human thymus exhibited features similar to those observed in mice, including impaired DNA replication, increased ECM-receptor interaction, and enhanced fatty acid metabolism-related activity, with thymic stromal cell analysis indicating that these processes are closely associated with mesenchymal cells.

**Conclusion:**

Increasing age disrupts early thymocyte differentiation and is accompanied by inflammatory-ECM remodeling and adipose-associated metabolic reprogramming. These integrative omics signatures nominate candidate pathways and regulators for developing interventions to mitigate ARTA and preserve immune homeostasis.

## Introduction

With increasing age, the immune system progressively exhibits hallmarks of immunosenescence and chronic low-grade inflammation (inflammaging), manifested as reduced anti-infectious and immune surveillance capacity and an elevated risk of inflammation-associated chronic diseases (Cisneros et al., 2022; Lewis et al., 2022; Ajoolabady et al., 2024). Among the diverse phenotypes of immune aging, declining thymic function is regarded as a key upstream event driving the contraction of the peripheral naïve T-cell pool and the reduction of T-cell diversity (Palmer et al., 2018; Lins and Dos Santos Reis, 2025).

The thymus is the central organ for T-cell development and the establishment of central tolerance. Thymocytes migrate through the cortico-medullary architecture and sequentially undergo the double-negative (DN), double-positive (DP), and single-positive (SP) stages. Cortical thymic epithelial cells (cTECs) primarily mediate positive selection, whereas medullary thymic epithelial cells (mTECs) and related antigen-presenting cells participate in negative selection and the maintenance of self-tolerance (Miller, 2020; Park et al., 2020; Ruiz Pérez et al., 2024). Age-related thymic atrophy (ARTA) is typically accompanied by reduced thymic size, cortical thinning, blurring of the corticomedullary boundary, impairment of thymic stromal and epithelial cell function, and varying degrees of adipose infiltration (Liang et al., 2022; Kousa et al., 2024; Lins and Dos Santos Reis, 2025). These changes weaken the sustained output of newly generated naïve T cells, thereby further compromising peripheral immune homeostasis and the quality of immune responses (Palmer et al., 2018; Liang et al., 2022).

ARTA is currently considered multifactorial, driven by the deterioration of the thymic stromal microenvironment, senescence and altered plasticity of thymic epithelial cells (TECs), accumulation of inflammatory signals, and metabolic reprogramming (Kousa et al., 2024; Lins and Dos Santos Reis, 2025). For example, thymic adipogenesis and altered cytokine availability (e.g., adipose tissue acting as an IL-7 “sink”) may directly affect thymopoiesis efficiency (Dooley and Liston, 2012). TEC-associated epithelial-mesenchymal transition (EMT) and an increased propensity for adipogenic differentiation have also been proposed to be closely linked to thymic structural remodeling (Zheng et al., 2023). However, systematic integrative evidence remains limited regarding which developmental checkpoints are first affected and how key transcriptional and proteomic pathways coordinately drive ARTA (Lins and Dos Santos Reis, 2025).

During thymocyte development, CD25 (Il2ra) and CD44 are commonly used to delineate DN subset differentiation trajectories and are closely associated with the development of thymic regulatory T cells (Treg) (Misra et al., 2015; Sun et al., 2023). Meanwhile, tolerance-related molecules such as Aire are essential for eliminating autoreactive thymocytes and maintaining immune tolerance (Peterson, 2026). Nevertheless, how these molecules change spatially within thymic regions during increasing age, and how such changes relate to developmental blockade and/or tolerance imbalance, remains to be further clarified.

Accordingly, using middle-age (MA) mice, we systematically evaluated alterations in thymic morphology, peripheral immune cell composition, and thymocyte developmental lineages, with a particular focus on the expression characteristics of CD25/CD44 across distinct thymic regions. By integrating transcriptomics and proteomics, we further delineated ARTA-associated molecular networks and pathway remodeling (e.g., inflammatory responses, extracellular matrix reorganization, and lipid metabolism), providing a basis for understanding the multifactorial mechanisms of age-related thymic atrophy and identifying potential intervention targets.

## Materials and methods

### Animals and ethical compliance

Specific-pathogen-free (SPF) male ICR mice were obtained from Hangzhou Medical College and assigned to a young group (1 month old, n = 12) and a middle-age group, which were purchased at 9 months and aged to 12 months before experiments. Animals were maintained under controlled conditions (20 °C–26 °C; 40%–70% humidity; 12 h light/12 h dark cycle; ventilation ≥22 air changes/hour) with *ad libitum* access to irradiated maintenance chow and sterilized drinking water. All animal experiments were approved by the Animal Ethics Committee of Zhejiang Shouxiangu Botanical Drug Institute (Approval No. 2024A01) and conducted in accordance with relevant guidelines and regulations for the care and use of laboratory animals.

### Thymus collection, gross assessment, and histology

Following euthanasia, thymuses were excised, photographed, and weighed. Tissues were fixed in paraformaldehyde (PFA), processed for paraffin embedding, sectioned, and used for routine hematoxylin-eosin (H&E) staining and immunohistochemistry (IHC).

### Peripheral blood cell analysis.

Peripheral blood was collected from the abdominal vein into EDTA-2K anticoagulant tubes, gently mixed, and analyzed (300 µL per sample) using an automated hematology analyzer (Sysmex XT-2000i).

### Flow cytometric analysis of T cell subtypes and proportions

The 100 μL of EDTA-K2-treated anti-coagulant peripheral blood was collected, and 2 μL of FITC-CD3 (Thermo, 11–0,032–82, clone 17A2), PE-CD4 (Biolegend, 100,407, clone GK1.5), and APC-CD8a (Biolegend, 100,711, clone 53–6.7) fluorescent antibodies were added successively, and incubated at room temperature (RT) for 15 min in the dark. Then add 1 mL erythrocyte lysate and incubate at RT for 15 min in the dark, centrifuge (500 g, 5 min) and discard the supernatant. Next, add 1 mL sterile PBS to wash precipitation, centrifuge (500 g, 5 min), and discard supernatant. Finally, 500 μL sterile PBS was added to resuspend precipitation, and the proportion of CD4^+^ and CD8^+^ T cells was measured using flow cytometry (CytoFLEX S, Beckman coulter life sciences), and data analysis was performed using CytExPert software.

### Oil Red O staining

Thymus tissues were fixed in 4% paraformaldehyde (Beyotime Biotechnology, P0099) and sequentially dehydrated in 15% and 30% sucrose solutions. Then, thymus tissues were embedded in OCT compound and sectioned into frozen sections. Next, the sections were rewarmed, fixed in 4% paraformaldehyde, and pre-differentiated with 60% isopropanol (Aladdin Biotechnology, I112011), followed by the application of Oil Red O staining solution (Beyotime Biotechnology, C0158). After rapid differentiation in 60% isopropanol, the sections were rinsed three times with distilled water. Finally, the sections were counterstained with hematoxylin (Hangzhou Haoke Biotechnology, HK 2053), blued in purified water, mounted, and the images were randomly captured by a microscope (DM4000, Leica Biosystems).

### TUNEL staining

Paraffin sections were deparaffinized in xylene (Sinopharm Chemical Reagent Co., 10,023,418) and rehydrated through graded ethanol including absolute ethanol (Hangzhou Hongda Chemical Instrument Co., SJ003614). After permeabilization with Proteinase K working solution (Beyotime Biotechnology, ST535), endogenous peroxidase activity was quenched by adding H_2_O_2_ (Aladdin Biotechnology, H112515). Subsequently, the sections were incubated with TdT reaction mixture (Beyotime Biotechnology, C1091), and the reaction was terminated using SSC solution, followed by sequential incubation with Streptavidin-HRP working solution and DAB chromogen solution. Next, the sections were counterstained with hematoxylin and blued in purified water. Finally, the sections were dehydrated through graded ethanol, cleared in xylene, and mounted with neutral resin (Sinopharm, 10,004,160). The images were randomly captured by a microscope.

### Immunofluorescence (IF)

Frozen thymus sections were rewarmed, fixed in 4% paraformaldehyde, permeabilized with 0.3% Triton X-100, and blocked with 5% bovine serum albumin (BSA; Hangzhou Haoke Biotechnology, HKW 2084).

The sections were incubated overnight at 4 °C with anti-CD3 (Proteintech, 17617-1-AP; 1:300), anti-CD4 (Proteintech, 19068-1-AP; 1:500), or anti-CD8 (Proteintech, 29896-1-AP; 1:300), followed by the corresponding CoraLite-conjugated goat anti-rabbit secondary antibodies (Proteintech, RGAR002 or RGAR004; 1:500). Nuclei were counterstained with DAPI. For quantification, three thymus tissues were randomly selected from each group, three non-consecutive sections were randomly collected from each thymus, and three non-overlapping fields were randomly captured from each section. Mean fluorescence intensity per field was quantified using ImageJ 1.41 software.

### Immunohistochemistry (IHC)

Paraffin sections were deparaffinized in xylene and rehydrated through graded ethanol including absolute ethanol. Antigen retrieval was performed using EDTA buffer (pH 8.0; Hangzhou Haoke Biotechnology, HKI0003), followed by washes in PBS (Hangzhou Haoke Biotechnology, HK0002). Endogenous peroxidase was quenched with 3% H_2_O_2_, and sections were blocked with 3% BSA.

For primary antibodies, sections were incubated overnight at 4 °C with anti-Ki-67 (Cell Signaling Technology, 12,202; 1:200), anti-CD44 (Abcam, ab243894; 1:500) or anti-CD25 (Cell Signaling Technology, 39,475; 1:200), followed by an HRP-conjugated goat anti-rabbit secondary antibody (Hangzhou Haoke Biotechnology, HKI0026). Color development was performed using DAB substrate (Proteintech, PR30010), and nuclei were counterstained with hematoxylin solutions (hematoxylin: Hangzhou Haoke Biotechnology, HK 2053; differentiation solution: HK 2054; bluing solution: HK 2055). Slides were dehydrated, cleared, and mounted with neutral resin, and the images were randomly captured by a microscope. For quantitative analysis of CD24 and CD44, three random fields of view were selected per section, the mean optical density of each field was analyzed using ImageJ 1.41 software (Bethesda, MD, USA), and the protein levels are expressed as fold-change in the MA group relative to the young group.

### Transcriptomic and proteomic profiling and analysis

Fresh thymus samples were used for RNA extraction and library construction, and libraries were sequenced on an Illumina NovaSeq 6,000 platform. Proteomic profiling was performed on an Orbitrap Astral high-resolution mass spectrometer using data-independent acquisition (DIA). Transcriptomic and proteomic experiments were conducted by LC Bio Technology Co., Ltd.

For transcriptomic data analysis, the raw expression matrix was processed as follows. Raw count data were first converted into a TPM (Transcripts Per Million) normalized expression matrix using the R package edgeR (v3.42.4). Differential expression analysis between groups was subsequently performed using DESeq2 (v1.42.0). Further downstream analyses included principal component analysis (PCA), Gene Ontology (GO) functional annotation, and Kyoto Encyclopedia of Genes and Genomes (KEGG) pathway enrichment.

For proteomic data analysis, the mass spectrometer was operated in data-dependent acquisition (DDA) PASEF mode. The capillary voltage was set to 4500 V. Spectra were acquired over m/z 100–1,700 Th and ion mobility range (1/K_0_) 0.7–1.3 V s/cm^2^. The ramp and accumulation time were 166 m, giving a total cycle time of 1.03 s. Collision energy was ramped linearly with mobility: from 59 eV at 1/K_0_ = 1.6 V s/cm^2^ to 20 eV at 1/K_0_ = 0.6 V s/cm^2^. Precursors with charge state 0–5 (charge 0 indicates that the isotope ion was not detected) were selected with a target intensity of 500 and an intensity threshold of 1,000. Dynamic exclusion was set to 0.4 min. The raw MS files were processed using PEAKS Online X (build 1.6.2022–01–07_161,036; Bioinformatics Solutions Inc.). Data were searched against the human protein database (UniProt, downloaded on [date]). Precursor mass tolerance was set to 20 ppm, and fragment mass tolerance to 0.05 Da. Trypsin was specified as the enzyme, allowing up to two missed cleavages. Carbamidomethylation of cysteine (+57.02 Da) was set as a fixed modification. Variable modifications included oxidation of methionine (+15.99 Da), phosphorylation of serine, and dehydration of serine. Peptide and protein identifications were filtered to a false discovery rate (FDR) < 1% at both levels.

In addition, in transcriptomic and proteomic analysis, multi-dimensional analyses were subsequently performed, including PCA-based dimensionality reduction and visualization, GO functional annotation, and KEGG pathway enrichment analysis.

### Analysis of translational relevance

Single-cell data of human thymus clinical samples were obtained from the GEO database (Gene Expression Omnibus) under accession number GSE231906. The dataset included 33 samples ranging from infancy to elderly. Based on established criteria (Deng et al., 2025), samples were divided into young (≤12 years, n = 11), middle (13–39 years, n = 9), and old (≥40 years, n = 13) groups. After aggregating the data into pseudo-bulk profiles, the activity of the DNA replication, ECM-receptor interaction, and fatty acid metabolism pathways was calculated for each sample using the single sample gene set enrichment analysis (ssGSEA) method. The classification and proportions of thymic stromal cells across different age groups were analyzed, and pathway activity in these cells was evaluated using the AUCell method.

### Statistical analysis

All data are presented as the mean ± standard error (SE) of at least three independent experiments. All data were first tested for D'Agostino-Pearson omnibus normality test. Unpaired two-tailed Student’s t-test was used for comparisons between two groups, and one-way analysis of variance (ANOVA) with Dunnett’s *post hoc* test was used for multiple comparisons. Specifically, in omics analysis, for differential expression analysis using DESeq2 in volcano plots, the Wald test is employed along with multiple comparison correction using the Benjamini–Hochberg method; for GO and KEGG enrichment analyses, the hypergeometric test is used; and for GSEA analysis, the permutation test is applied. The P < 0.05 was considered statistically significant.

## Results

### Increasing age is associated with age-related thymic atrophy (ARTA) and impaired T-cell output

**FIGURE 1:**
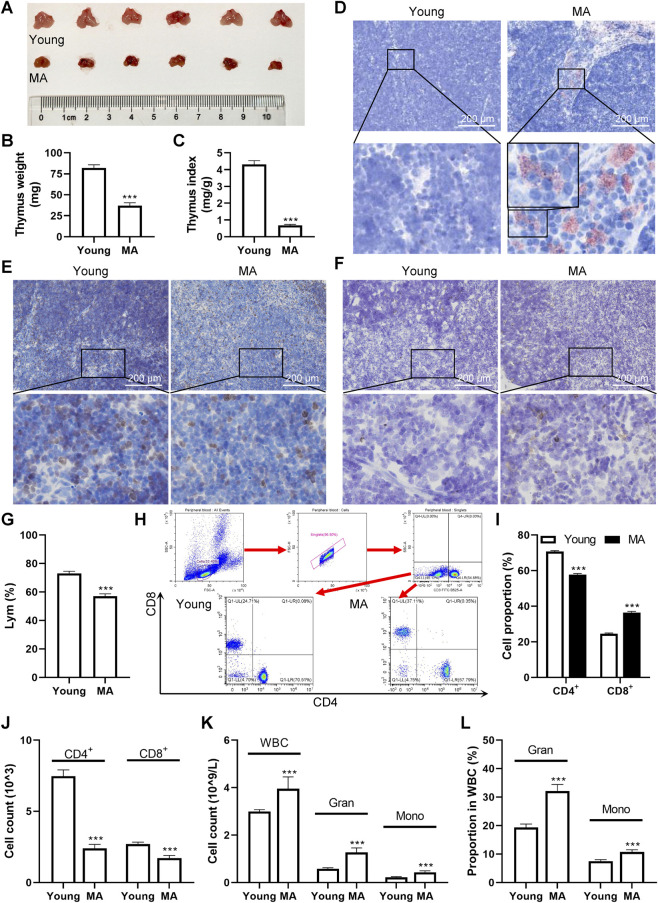
Increasing age-associated thymic atrophy impairs T-cell output. **(A–C)** Representative images of the thymus **(A)**, thymus weight **(B)** and thymic index **(C)**. **(D–F)** Representative images of Oil Red O staining **(D)** immunohistochemical staining (Ki-67) **(E)** and TUNEL staining **(F)** of thymic tissue (Scale bar = 200 μm). **(G)** Percentage of lymphocytes in peripheral blood. **(H,I)** Flow cytometric analysis of the frequencies of CD4^+^ and CD8^+^ lymphocytes in peripheral blood **(H)** and quantification **(I)**. **(J)** Absolute numbers of CD4^+^ and CD8^+^ lymphocytes in peripheral blood. **(K)** Absolute numbers of total leukocytes, neutrophils, and monocytes in peripheral blood. **(L)** Proportions of neutrophils and monocytes among total leukocytes. Data were analyzed by unpaired two-tailed Student’s t-test, normality was confirmed by D’Agostino-Pearson omnibus normality test. Compared with the Young group, ***P < 0.001; n = 6–12.

Changes in thymic mass, apoptosis of thymic epithelial cells (TECs), and adipocyte infiltration are among the most prominent features of ARTA. As shown in Figures 1A–C, compared with the young (Young) group, mice in the middle-age (MA) group exhibited a marked reduction in thymic mass (Figure 1A), including decreased thymic weight (Figure 1B) and thymic index (Figure 1C). Oil Red O staining revealed increased lipid droplet accumulation in thymic tissue from the MA group compared with the Young group (Figure 1D). Immunohistochemistry and TUNEL staining further revealed that Ki-67 expression, a marker of cell proliferation, was decreased (Figure 1E), whereas the proportion of apoptotic TECs was increased (Figure 1F) in thymic tissue from the MA group. The thymus is the primary site for T-cell development; therefore, thymic involution inevitably compromises T-cell maturation. As shown in Figure 1G, the proportion of lymphocytes in peripheral blood was significantly reduced in MA mice compared with Young controls. We next analyzed T-cell subsets and frequencies in peripheral blood. As shown in Figures 1H,I, the frequency of CD3^+^CD4^+^ T cells was significantly decreased, whereas the frequency of CD3^+^CD8^+^ T cells was significantly increased in MA mice. However, the absolute numbers of both CD3^+^CD4^+^ and CD3^+^CD8^+^ T cells were significantly reduced (Figure 1J).

In addition to lymphopenia, increased myelopoiesis is another key feature of immune aging (Ruffinatto et al., 2024). As shown in Figure 1K, compared with Young controls, MA mice displayed significantly increased counts of white blood cells, neutrophils, and monocytes in peripheral blood, and the proportions of neutrophils and monocytes among total leukocytes were also significantly elevated (Figure 1L). Collectively, these findings indicate that MA induces ARTA and promotes immune senescence.

### Increasing age-associated ARTA impairs thymocyte development

**FIGURE 2:**
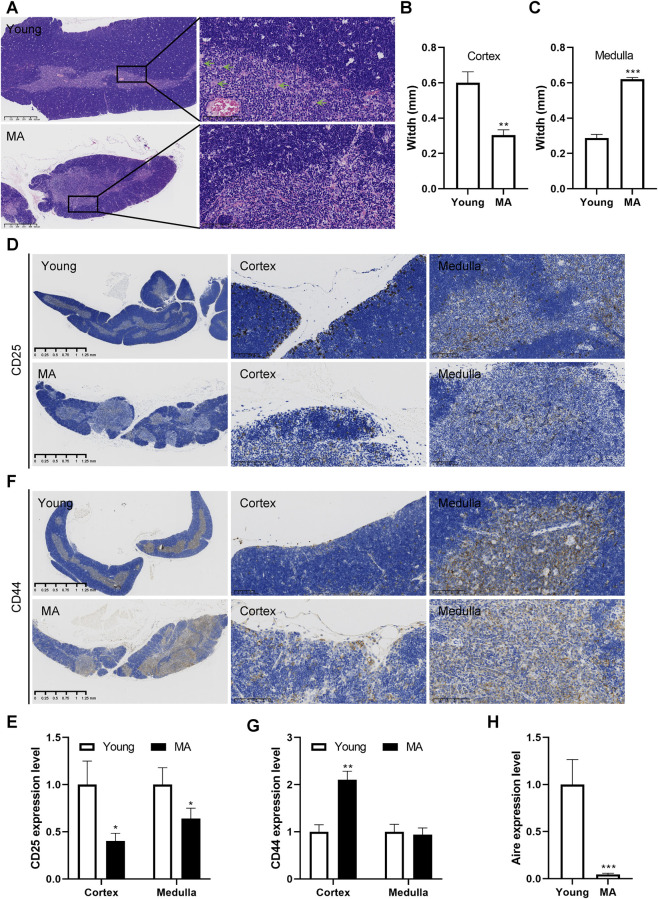
Increasing age-associated thymic atrophy disrupts thymocyte development. **(A–C)** Representative H&E-stained images of thymic sections **(A)** and quantitative analysis of cortical **(B)** and medullary **(C)** thickness. **(D,E)** Immunohistochemical staining of CD25 in thymic tissue **(D)** and semi-quantification in the cortex and medulla **(E)**. **(F,G)** Immunohistochemical staining of CD44 in thymic tissue **(F)** and semi-quantification in the cortex and medulla **(G)**. **(H)** Aire protein expression in thymic tissue. Data were analyzed by unpaired two-tailed Student’s t-test, normality was confirmed by D’Agostino-Pearson omnibus normality test. Compared with the Young group, *P < 0.05, **P < 0.01, ***P < 0.001; n = 3–6.

Morphologically, the thymus is a reticular organ composed of a cortex and a medulla, and thymic atrophy is primarily characterized by spatial and structural alterations in these compartments. We therefore examined histological changes in the thymus of MA mice. As shown in Figure 2A, the thymic architecture of Young mice was intact, with a clear corticomedullary boundary: the cortex contained densely packed thymocytes, and the medulla exhibited characteristic thymic corpuscles (Hassall’s corpuscles). In contrast, the thymus of MA mice showed disorganized architecture with a blurred corticomedullary boundary; cortical thickness was markedly reduced (Figure 2B), whereas medullary thickness was increased (Figure 2C), suggesting that increasing age-associated ARTA may disrupt thymocyte development within cortical and medullary regions.

The cortex is the site of TCRβ rearrangement and positive selection of double-negative (DN) thymocytes, which progress through four stages: DN1 (CD25^−^CD44^high^) → DN2 (CD25^+^CD44^+^) → DN3 (CD25^+^CD44^−^) → DN4 (CD25^−^CD44^−^). As shown in Figures 2D–G, compared with Young controls, CD25 expression was significantly reduced (Figures 2D,E) and CD44 expression was significantly increased (Figures 2F,G) in the thymic cortex of MA mice, suggesting a potential developmental blockade at the DN1-to-DN2 transition. In addition, the medulla is the site of negative selection of single-positive (SP) thymocytes, and we observed significantly reduced CD25 expression in the medullary region of MA mice (Figures 2D,E). CD25 is typically highly expressed on naturally occurring regulatory T cells (nTreg; CD4^+^CD25^+^), which are central mediators of peripheral immune tolerance. Thus, decreased CD25 expression may contribute to reduced Treg output. Notably, the expression of Aire (semi-quantitative results are derived from proteomic analysis) was also markedly decreased in the thymus of MA mice (Figure 2H). Aire promotes central tolerance by driving the expression of tissue-restricted antigens in the thymus, thereby enabling SP thymocytes to acquire self-tolerance and preventing autoimmune disease.

**FIGURE 3:**
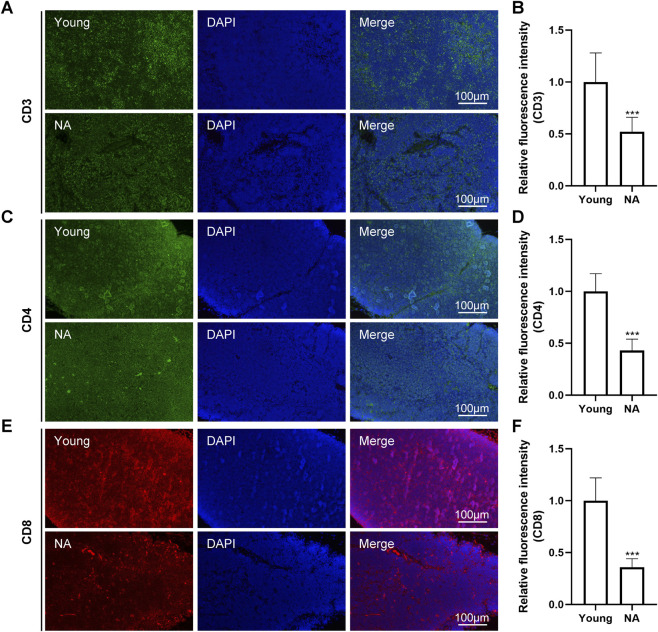
Analysis of thymocyte subsets and proportions. **(A–F)** Immunofluorescence staining showing the proportions of CD3^+^ **(A)** CD4^+^ **(C)** and CD8^+^ **(E)** thymocytes in thymic tissue, with semi-quantification **(B,D,F)**. Data were analyzed by unpaired two-tailed Student’s t-test, normality was confirmed by D’Agostino-Pearson omnibus normality test. Compared with the Young group, ***P < 0.001; n = 3.

To further define the impact of natural aging on thymocyte development, we further assessed the proportions of CD3+, CD4+, and CD8+ thymocytes in thymic sections by immunofluorescence. As shown in Figures 3A–F, compared with controls, ARTA mice exhibited significant reductions in CD3+, CD4+, and CD8+ thymocytes. Together, these results suggest that ARTA may impair thymocyte development by blocking the DN1-to-DN2 transition and disrupting selection processes of thymocytes, including the DP stage.

### Increasing age-associated thymic atrophy involves impaired DNA replication, heightened inflammation, and upregulated lipid metabolism

**FIGURE 4:**
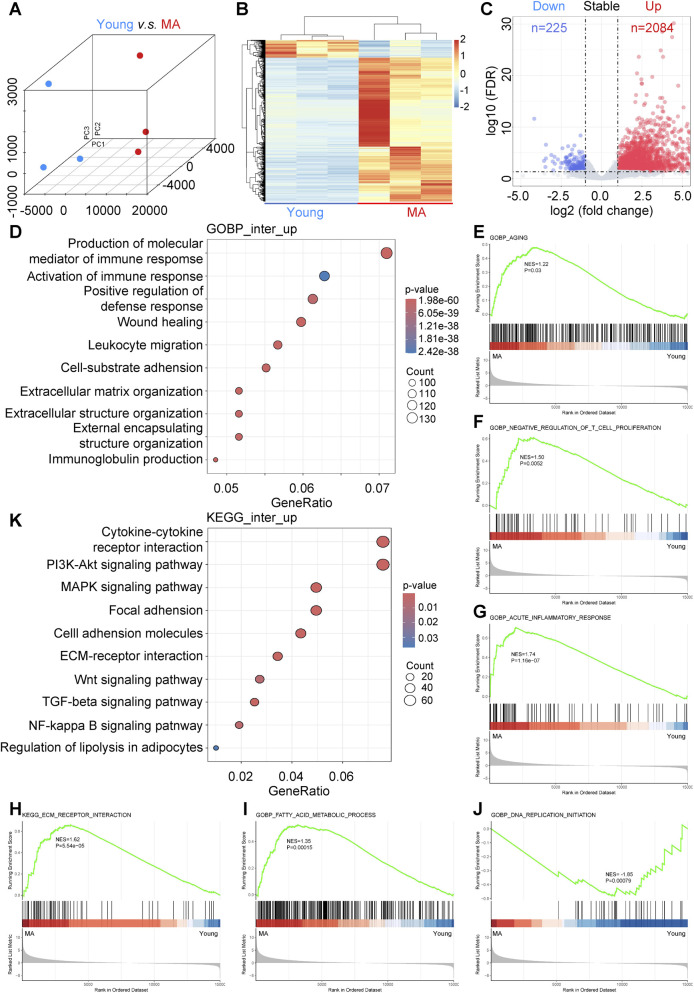
Transcriptomic profiling of increasing age-associated thymic atrophy. **(A–C)** PCA **(A)** clustering analysis **(B)** and volcano plot of differentially expressed genes **(C)**. **(D)** GO Biological Process (GOBP) enrichment analysis of significantly upregulated genes. **(E–J)** GSEA for Aging, Negative regulation of T cell proliferation, Acute inflammatory response, ECM-receptor interaction, Fatty acid metabolic process, and DNA replication initiation. **(K)** KEGG pathway enrichment analysis of significantly upregulated genes. For differential expression analysis using DESeq2 in volcano plots, the Wald test is employed along with multiple comparison correction using the Benjamini–Hochberg method; for GO and KEGG enrichment analyses, the hypergeometric test is used; and for GSEA analysis, the permutation test is applied. Note: all RNA-seq samples in this study were processed in the same experimental batch: RNA extraction, library preparation, and sequencing were performed on the same platform under identical experimental conditions. Therefore, no formal batch-effect correction was applied, and no outlier samples were excluded from the analysis. The percentages of variance explained by PC1 and PC2 were 42.39% and 23.46%, respectively.

To elucidate the regulatory mechanisms underlying increasing age-associated thymic atrophy, we performed transcriptomic profiling. Principal component analysis (PCA; Figure 4A) and clustering analysis (Figure 4B) revealed pronounced transcriptomic changes in thymic tissues from MA mice. Compared with the Young group, 2,084 genes were significantly upregulated and 255 genes were significantly downregulated in the MA group (Figure 4C). Gene Ontology (GO) biological process enrichment indicated that the differentially expressed genes were primarily associated with immune-related processes (Figure 4D). Gene set enrichment analysis (GSEA) showed significant upregulation of gene signatures related to aging (Figure 4E), negative regulation of T-cell expansion (Figure 4F), inflammatory responses (Figure 4G), ECM-receptor interaction (Figure 4H), and fatty acid metabolism (Figure 4I), whereas DNA repair-related gene signatures were significantly downregulated in aged thymic tissue (Figure 4J). These results suggest that aging may weaken thymic function by promoting inflammation and suppressing DNA replication-related programs (Pangrazzi and Weinberger, 2020; Mittelbrunn and Kroemer, 2021), thereby contributing to defects in T-cell development and expansion. Consistently, KEGG analysis indicated enrichment of multiple pathways implicated in thymocyte development or survival—such as cytokine-cytokine receptor interaction, cell adhesion molecules, MAPK signaling, Wnt signaling, TGF-β signaling, ECM-receptor interaction, and PI3K-Akt signaling—in aged thymic tissue (Figure 4K).

Notably, GO enrichment highlighted three distinctive biological processes—wound healing, leukocyte migration, and extracellular matrix organization (Figure 4D). Recent studies have shown that following skin injury, neutrophils rapidly accumulate around the wound and continuously secrete extracellular matrix components to facilitate wound repair, a process driven by TGF-β signaling (Vicanolo et al., 2025). These findings imply that the neutrophilia accompanying immune senescence may reflect a TGF-β-dependent self-repair response in the damaged thymus.

### Increasing age induces thymic adipose replacement

**FIGURE 5:**
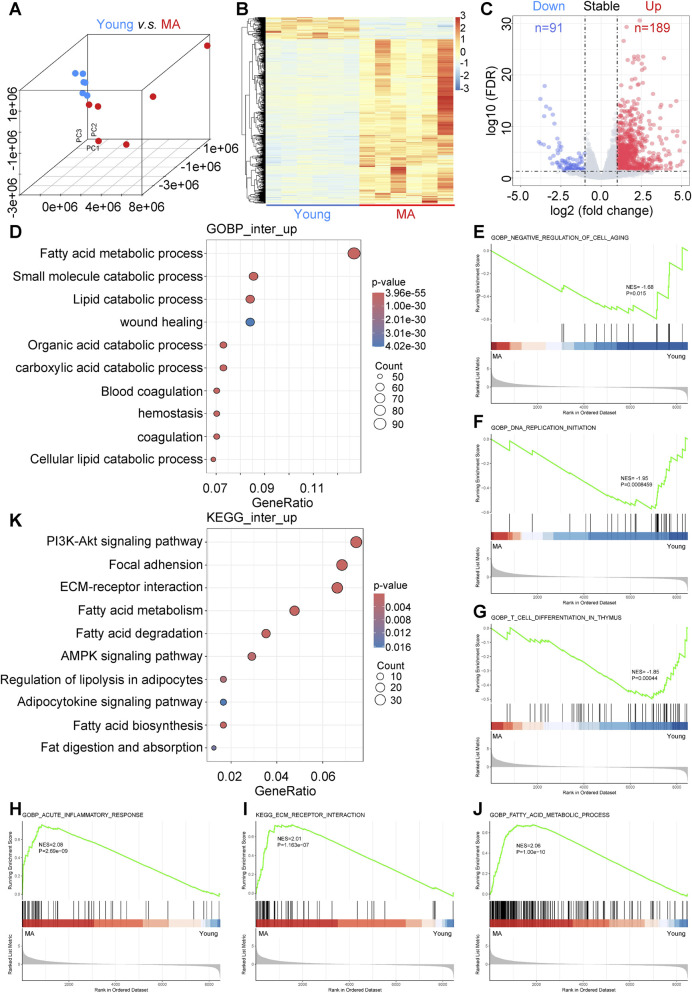
Proteomic profiling of increasing age-associated thymic atrophy. **(A–C)** PCA **(A)** clustering analysis **(B)** and volcano plot of differentially expressed proteins **(C)**. **(D)** GOBP enrichment analysis of significantly upregulated proteins. **(E–J)** GSEA for Negative regulation of cell aging, DNA replication initiation, T cell differentiation in thymus, Acute inflammatory response, ECM-receptor interaction, and Fatty acid metabolic process. **(K)** KEGG pathway enrichment analysis of significantly upregulated proteins. For differential expression analysis using DESeq2 in volcano plots, the Wald test is employed along with multiple comparison correction using the Benjamini–Hochberg method; for GO and KEGG enrichment analyses, the hypergeometric test is used; and for GSEA analysis, the permutation test is applied.

Because proteins are the direct effectors of biological function, we performed proteomic profiling to further investigate potential mechanisms of age-associated thymic atrophy. PCA (Figure 5A) and clustering analysis (Figure 5B) revealed substantial alterations in the thymic proteome of middle-age mice, indicating proteomic remodeling induced by increasing age. Compared with Young controls, 189 proteins were significantly upregulated and 91 proteins were significantly downregulated in aged thymic tissue (Figure 5C). GO biological process enrichment showed that the altered proteins were mainly associated with metabolic processes, including fatty acid metabolic process, small-molecule catabolic process, and lipid catabolic process (Figure 5D), consistent with the phenotype of adipose replacement in the aged thymus. GSEA further demonstrated that proteins involved in negative regulation of cellular senescence (Figure 5E), DNA replication (Figure 5F), and thymic T-cell differentiation (Figure 5G) were decreased, whereas proteins related to inflammatory responses (Figure 5H), ECM-receptor interaction (Figure 5I), and fatty acid metabolism (Figure 5J) were increased. This highlights a physiological degenerative trajectory of thymic atrophy characterized by adipose replacement, inflammation, functional decline, and aberrant T-cell development (Mittelbrunn and Kroemer, 2021). KEGG analysis indicated that the differentially expressed proteins were primarily involved in cellular signaling (focal adhesion and ECM-receptor interaction) and energy metabolism (fatty acid metabolism, fatty acid degradation, and the AMPK signaling pathway), and these pathways may be regulated by PI3K/Akt signaling (Koundouros and Poulogiannis, 2020; Wang et al., 2024; Fang et al., 2025) (Figure 5K). Collectively, these results suggest that increasing age drives the thymus to degenerate from a lymphoid organ dominated by immune cells into a tissue composed of mixed adipose and immune cell populations (Li and Zúñiga-Pflücker, 2023).

### Aberrant ECM remodeling and chronic inflammation jointly drive age-related thymic involution

**FIGURE 6:**
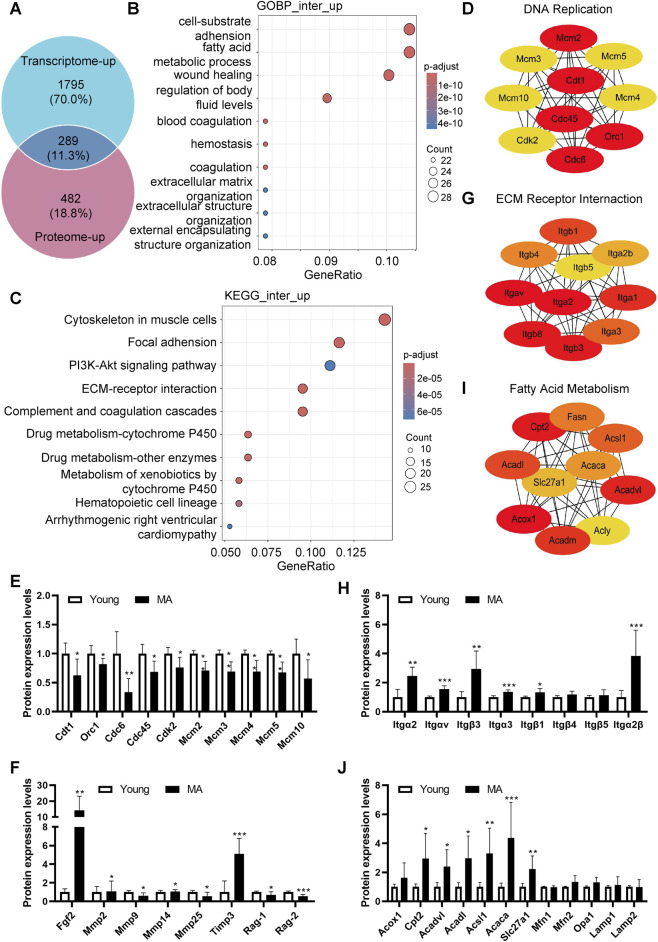
Multi-omics analyses reveal that aberrant ECM remodeling and chronic inflammation drive increasing age-associated thymic atrophy. **(A)** Venn diagram of coordinately upregulated gene-protein pairs. **(B,C)** GOBP and KEGG enrichment analyses of coordinately upregulated gene-protein pairs. **(D,E)** Regulatory network related to DNA replication **(D)** and expression levels of the associated proteins in thymic tissue **(E)**. **(F)** Expression levels of proteins involved in extracellular matrix remodeling and key proteins for TCRα-chain rearrangement in the thymus. **(G,H)** Regulatory network related to ECM-receptor interaction **(G)** and expression levels of integrin proteins **(H)**. **(I,J)** Regulatory network related to fatty acid metabolism **(I)** and expression levels of proteins associated with fatty acid metabolism and lysosomal function **(J)**. Data were analyzed by unpaired two-tailed Student’s t-test, normality was confirmed by D’Agostino-Pearson omnibus normality test. Compared with the Young group, *P < 0.05, **P < 0.01, ***P < 0.001; n = 6.

To more comprehensively delineate the gene-protein regulatory landscape underlying increasing age-induced thymic atrophy, we performed an integrative analysis of transcriptomic and proteomic datasets. As shown in Figure 6A, 289 gene-protein pairs were coordinately upregulated, and these upregulated pairs were mainly enriched in biological processes such as cell-substrate adhesion, fatty acid metabolic process, and wound healing (Figure 6B). KEGG analysis further indicated that these gene-protein pairs predominantly mapped to pathways including cytoskeleton in muscle cells, focal adhesion, PI3K/Akt signaling, and ECM-receptor interaction (Figure 6C). These findings suggest that the aged thymic parenchyma is progressively infiltrated/replaced by adipose tissue and that negative selection mediated by medullary thymic epithelial cells (mTECs) through cell-cell interactions may be disrupted. Notably, complement and coagulation cascades were enriched by KEGG analysis (Figure 6C), representing proteolytic cascades involving the complement and coagulation systems. On the one hand, complement activation generates the membrane attack complex (MAC, C5b-9), whose deposition on thymic epithelial cells can directly compromise the structure and function of the thymic microenvironment (Leite et al., 2007). On the other hand, activation of protease-activated receptor (PAR) signaling within the coagulation system can promote apoptosis of thymocytes and thymic epithelial cells (Tena-Chaves et al., 2025), thereby exacerbating deterioration of the thymic niche. Importantly, both systems are key drivers of autoimmune disease initiation and progression (Xie et al., 2020; Xu et al., 2024).

Subsequently, guided by the GSEA results, we further interrogated key factors involved in aging, DNA replication, T-cell differentiation, inflammatory responses, ECM-receptor interaction, and fatty acid metabolism.

Cellular aging is often accompanied by cell-cycle arrest and defects in DNA replication (Ogrodnik, 2021). During DNA replication, Cdc10-dependent transcript 1 (CDT1) and Cdc45 play central regulatory roles (Figure 6D). In early G1 phase, Orc1 recruits Cdc6 and Cdt1 to replication origins and loads the MCM helicase complex to ensure orderly cell-cycle progression (Hossain et al., 2021). During S phase, Cdc45 associates with the replication complex, including Go-Ichi-Ni-San (GINS) and MCM, to activate helicase activity and drive DNA synthesis (Lim et al., 2023). However, the levels of key proteins involved in cell-cycle control and DNA replication—such as Cdc45, CDT1, Orc1, and Cdc6—were significantly reduced in the thymus of NA mice (Figure 6E), indicating that regenerative capacity of the aged thymus is severely compromised.

Inflammatory activation and extracellular matrix degradation are hallmark features of thymic involution (Ruiz Pérez et al., 2024). Fgf2, a proposed bridge linking inflammation and aging, was highly expressed in the thymus of MA mice (Figure 6F). Fgf2 may aggravate inflammation and accelerate aging by promoting IL-1β and IL-6 expression, a process accompanied by ECM degradation (Song et al., 2021). However, we did not observe a significant increase in MMP expression in MA thymic tissue (Figure 6F). Instead, expression of Timp3, an endogenous inhibitor of MMPs, was significantly elevated (Figure 6F), which may represent a compensatory response to extensive ECM degradation within the thymic microenvironment. Nevertheless, the expression of recombination activating genes Rag1 and Rag2—key regulators of TCRα-chain rearrangement—was significantly decreased in the MA group, suggesting that T-cell repertoire diversity may be compromised (Nishana and Raghavan, 2012).

In the regulation of ECM-receptor interaction, multiple members of the integrin family play critical roles (Kanchanawong and Calderwood, 2023). Previous studies indicate that integrin signaling spans the entire course of thymocyte development, including progenitor homing, TCRβ-chain rearrangement, positive selection, negative selection, and thymic egress (Moretti et al., 2018; Potter et al., 2024; Liman et al., 2025). As shown in Figures 6G,H, compared with the Young group, expression of multiple integrins was significantly increased in the thymus of MA mice, which may strengthen interactions between thymocytes and stromal cells. However, excessively strong adhesion may hinder thymocyte migration within the thymic microenvironment (Jeffreys et al., 2025). Moreover, enhanced integrin signaling may facilitate adipocyte recognition, attachment, and microenvironmental remodeling within thymic tissue (Dixit, 2010; Liang et al., 2022; Lins and Dos Santos Reis, 2025). Together, these data suggest that integrin signaling exerts dual roles in thymocyte development and age-associated thymic degeneration.

Within fatty acid metabolism, several regulators of long-chain fatty acid metabolism were enriched, including Acox1, Cpt2, Acadvl, Acadl, Acsl1, Acaca, and Slc27a1 (Figure 6I). Interestingly, the abundance of these proteins was significantly increased in the thymus of aged mice (Figure 6J), which appears inconsistent with the commonly described phenotype of increasing age-associated lipid metabolic dysregulation. This pattern may indicate a shift in metabolic dominance from thymic epithelial cells to adipocytes as adipose tissue accumulates within the thymus. Because mitochondria are the primary site of long-chain fatty acid oxidation, we next assessed changes in mitochondrial function. As shown in Figure 6J, compared with Young controls, key proteins involved in mitochondrial dynamics (Mfn1, Mfn2, and Opa1) and lysosome-associated/mitophagy-related function (Lamp1 and Lamp2) did not change significantly in MA thymic tissue. These findings suggest that enhanced long-chain fatty acid metabolism in the aged thymus may reflect increased fatty acid metabolism-related activity, despite no marked changes in selected markers of mitochondrial dynamics, and is consistent with degenerative replacement of thymic parenchyma by adipose tissue.

### Mesenchymal cells are associated with age-related thymic remodeling in humans

**FIGURE 7:**
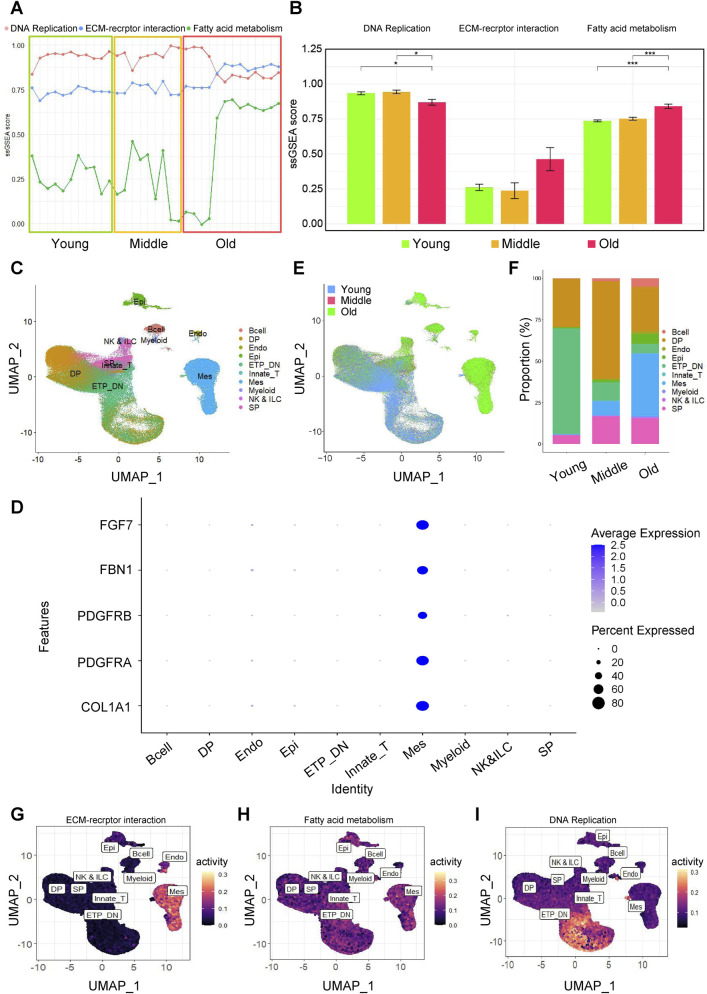
Analysis of age-related thymic changes in humans. **(A,B)** SsGSEA of DNA replication, ECM-receptor interaction, and fatty acid metabolism pathways in human thymic tissues across different age groups **(A)**, with corresponding quantification shown in **(B)**. **(C)** UMAP plot showing the classification of thymic stromal cells. **(D)** Signature genes of Mes cells. **(E,F)** UMAP plots showing the distribution of thymic stromal cells across different age groups **(E)**, with corresponding quantification in **(F)**. **(G–I)** Pathway activity analysis of ECM-receptor interaction **(G)**, fatty acid metabolism **(H)**, and DNA replication **(I)** in thymic stromal cells across different age groups. Data were analyzed by one-way ANOVA followed by Dunnett’s *post hoc* test, normality was confirmed by D’Agostino-Pearson omnibus normality test. *P < 0.05, ***P < 0.001; n = 9–13.

Translating basic research into clinical applications is one of the important goals of scientific research. However, the similarities and differences in ARTA between mice and humans remain unclear. Therefore, we analyzed single-cell data from human thymus samples obtained from the GEO database. The results showed that the activities of DNA replication, ECM-receptor interaction, and fatty acid metabolism pathways in thymic tissue remained stable within a certain range before the age of 40 (Figures 7A,B). However, after the age of 40, the activity of DNA replication gradually decreased, while the activities of ECM-receptor interaction and fatty acid metabolism gradually increased (Figures 7A,B), a pattern similar to that observed in aged mouse thymus. Subsequently, we analyzed the classification and proportions of thymic stromal cells (Figure 7C) and found that the proportion of mesenchymal cells [Mes, marker genes include COL1A1, PDGFRA, PDGFRB, FBN1, and FGF7 (Deng et al., 2025)] in thymic tissue increased significantly with age (Figures 7D-F), and these mesenchymal cells exhibited active ECM-receptor interaction (Figure 7G) and fatty acid metabolism (Figure 7H), along with weak DNA replication activity (Figure 7I). These findings suggest that mesenchymal cells may serve as adipocyte precursors in the thymus and may contribute to age-related thymic remodeling.

## Discussion

Increasing age is a systemic and progressive decline in organismal function. Organ atrophy is not only one of the most intuitive morphological manifestations of Increasing age, but also a core foundation for many physiological and pathological changes. In most organs, atrophy and functional deterioration typically begin in middle and older age; however, the thymus—the primary site of T-cell development—starts to involute as early as puberty, making thymus-dependent immunity one of the earliest age-associated events (Kologrivova et al., 2024). With advancing age, the thymus undergoes irreversible shrinkage, architectural disruption, and functional decline, referred to as age-related thymic atrophy (ARTA). ARTA is a complex physiological process involving hormonal, cellular, and molecular interactions, ultimately leading to reduced mature T-cell output and defects in the immune repertoire (Deng et al., 2025). Nevertheless, the intrinsic regulatory mechanisms of ARTA and its impact on thymocyte development remain incompletely understood. In this study, we analyzed the pathological changes of the aged thymus *in vivo* and their consequences for thymocyte development, and further leveraged multi-omics profiling to reveal multifactorial mechanisms underlying ARTA.

During T-cell development, thymocytes migrate within the thymic reticular framework and progress through the double-negative (DN), double-positive (DP), and single-positive (SP) stages. TCRα rearrangement in DP thymocytes and their subsequent interaction with cortical thymic epithelial cells (positive selection) define the main developmental axis, whereas interactions between medullary thymic cells and SP thymocytes (negative selection) ensure appropriate thymocyte export (Kondo et al., 2019). However, factors such as hormones, Increasing age, and infection can disrupt thymic structure; collapse of the three-dimensional reticular network can impede thymocyte migration and development (Luo et al., 2021; Li and Zúñiga-Pflücker, 2023; Yang et al., 2024). In our study, the thymus exhibited prominent pathological features including cortical narrowing, blurring of the cortico-medullary boundary, and reduced cellularity in MA mice, and the concomitant reduction in peripheral CD4^+^ and CD8^+^ T cells supports the notion that these structural changes compromise thymocyte development. During negative selection, Aire eliminates autoreactive thymocytes by regulating tissue-restricted antigen expression, thereby preventing autoimmune disease (Miller et al., 2024). Here, Aire expression was significantly reduced in the aged thymus and was accompanied by decreased CD25 protein levels in the medullary region, implying that both Aire-mediated central tolerance and Treg-associated peripheral tolerance may be compromised—potentially contributing to the increased incidence of autoimmune diseases in older individuals (Abend et al., 2024). In addition, integrin signaling is essential for thymocyte-stromal adhesion and signal transduction (Evans et al., 2009; Pang et al., 2023). Importantly, appropriate thymocyte-stromal affinity must fall within an optimal range to support normal development, as excessively high or low affinity can lead to deletion (Luan et al., 2019). We found that multiple integrins were significantly upregulated in the aged thymus, which may be unfavorable for thymocyte selection and migration within the thymic microenvironment; alternatively, this could reflect a compensatory strengthening of thymocyte-stromal interactions secondary to reduced T-cell output.

Although lymphocytes mature in the thymus, their origin is the bone marrow rather than the thymus. Under physiological conditions, bone marrow-derived hematopoietic stem cells transition from a quiescent (qHSC) to an activated state (aHSC) and differentiate into lymphoid, myeloid, and erythroid lineages (Ruffinatto et al., 2024). Aging or inflammatory states can trigger sustained HSC reprogramming with a bias toward myeloid differentiation, resulting in reduced lymphopoiesis (Ross et al., 2024; Ruffinatto et al., 2024). Consistently, we observed decreased lymphocytes in peripheral blood of ARTA mice, accompanied by increased myeloid cells (neutrophils and monocytes). Terekhova et al. proposed that sustained HSC reprogramming may represent a reparative shift to a new equilibrium characterized by high inflammation and low regenerative capacity, following thymus-driven immune imbalance (Terekhova et al., 2025). In our multi-omics analysis of the aged thymus, enrichment of biological processes such as wound healing, leukocyte migration, and extracellular matrix (ECM) organization suggests that myeloid cell recruitment to the thymus might help mitigate tissue injury, although direct evidence remains limited. Notably, recent work reported that TGF-β signaling can drive neutrophils to acquire ECM-related features and migrate to injured skin, where they secrete collagen and promote wound repair (Vicanolo et al., 2025). In addition, we detected a significant increase in Timp3 protein in the aged thymus. In a bleomycin-induced acute lung injury model, Timp3^(−/−)^ mice exhibited persistent inflammation and increased pulmonary MMP activity, whereas MMP inhibitors accelerated inflammatory resolution (Gill et al., 2010). Given that Timp3 is highly expressed in myeloid cells (Tabula Muris Consortium, Overall coordination, Logistical coordination, Organ collection and processing, Library preparation and sequencing, Computational data analysis, Cell type annotation, Writing group, Supplemental text writing group, Principal investigators, 2018), these observations support the hypothesis that myeloid cell trafficking to the thymus may help attenuate inflammation and restrain ECM degradation, thereby facilitating restoration of thymic function.

Adipocyte infiltration and thymic adipose involution are among the hallmark late-stage features of ARTA. By ∼45 years of age in humans, adipose tissue composed of lipid-rich, pro-inflammatory multilocular adipocytes can occupy ∼75% of thymic volume (Youm et al., 2012). However, the cellular origin of these adipocytes remains debated. One model suggests that mesenchymal stem cells and pericytes in perivascular spaces differentiate into adipocytes through activation of PPARγ, and that caloric restriction reduces PPARγ expression and suppresses thymic adipose degeneration (Hu et al., 2021). Another model proposes that TGF-β signaling induces an interaction between CD147 (EMMPRIN; an MMP inducer) expressed on T cells and thymic epithelial cells, driving epithelial-to-mesenchymal transition (EMT) of thymic epithelial cells toward a mesenchymal stem-like state and subsequent adipogenic differentiation (Chen et al., 2021; Liang et al., 2022). Intriguingly, thymectomy significantly reduces CD4 and CD8 lymphocyte formation, increases circulating pro-inflammatory factors, and elevates all-cause mortality (including cancer-related mortality) (Kooshesh et al., 2023), suggesting that an adipose-involuted thymus may retain limited functional relevance to immune homeostasis (Cavallotti et al., 2008). Thymic function is reported to depend strongly on the adipose-derived cytokine leptin (Kellogg and Equils, 2021), which alleviates lipopolysaccharide/inflammation-induced thymocyte apoptosis by inhibiting p38 MAPK activation and regulates the thymocyte developmental axis (Liang et al., 2013; Orlova et al., 2023), and leptin-deficient mice show severe thymic atrophy (Hick et al., 2006), consistent with the hypothesis that thymic adipocytes may exert paracrine effects on thymocytes and/or thymic epithelial cells (Cavallotti et al., 2008). A well-established metabolic action of leptin is to promote AMPK-dependent fatty acid β-oxidation (Kim et al., 2023). In this study, despite no marked changes in selected markers of mitochondrial dynamics and lysosome-associated function, fatty acid metabolism-related activity was increased in the aged thymus, primarily reflected by higher expression of key enzymes involved in fatty acid β-oxidation. Specifically, upregulating PPAR-mediated fatty acid metabolism ameliorates glucocorticoid-induced thymic atrophy and promotes thymocyte development (Li et al., 2018), implying that adipose involution could represent a compensatory mechanism triggered by thymic functional decline. Similarly, this compensatory mechanism may also exist in tumor-induced thymic atrophy (Lamas et al., 2016). Interestingly, co-culturing thymocytes with adipocytes derived from tumor-bearing mice promotes the production of IL-2 and IFN-γ by thymocytes, which facilitate the development of Treg and CD8^+^ T cells, respectively (Kanamori et al., 2016; Etzensperger et al., 2017). Nevertheless, metabolic compensation may still fail to prevent progression of inflammation, as also observed in our data; therefore, controlling inflammation may be more beneficial for compensatory recovery of thymic function.

In the single-cell data of human thymus samples, we found that mesenchymal cells exhibit characteristics highly consistent with those in the mouse thymus, including diminished DNA replication activity and enhanced ECM-receptor interaction and fatty acid metabolism. These findings are consistent with both hypotheses mentioned above, suggesting that mesenchymal cells may localize to thymic tissue and differentiate into adipocytes through certain mechanisms. Notably, leptin activates the LepR/JAK2/AMPK signaling pathway in adipocytes to promote mitochondrial biogenesis and fatty acid transport into mitochondria, a process that is independent of mitochondrial fusion/fission (Zhang et al., 2020). Therefore, we observed no significant changes in the expression of genes related to mitochondrial dynamics in the MA group. More interestingly, in both humans and mice, Postn^+^ mesenchymal cells promote adult thymic regeneration and enhance *de novo* T cell generation and immune responses to vaccination by producing the chemokine Ccl19 (Gustafsson et al., 2025). Conversely, specific knockout of neural-crest-derived mesenchymal cells reduces the number of DN1-like early T cell progenitors and impairs thymic regenerative capacity (Tetteh et al., 2025). These studies highlight the important role of mesenchymal cells in regulating thymic regeneration and immunity. In our study, we found enhanced ECM-receptor interaction in mesenchymal cells, suggesting that this may represent an intrinsic but insufficient self-repair response within the gradually involuting thymic microenvironment.

In summary, ARTA is a degenerative process driven by multiple factors including Increasing age, inflammation, and adipose involution, and alleviating increasing age-associated damage and inflammation may help mitigate ARTA. Moreover, during ARTA progression, the immune system may maintain systemic immune homeostasis and thymic self-repair by reprogramming HSC differentiation. However, several limitations of this study should be acknowledged: (1) This study only analyzed thymus changes from young to middle-aged mice, lacking data from aged mice. In future studies, we will include 18-month-old and 24-month-old mice to verify whether the molecular features identified in this study persist or further intensify during advanced thymic aging. (2) This study did not directly analyze the impact of ARTA on DN thymocyte development using techniques such as flow cytometry, immunofluorescence, or immunohistochemistry. (3) Although thymus-associated adipocytes may represent a potential target for improving ARTA-associated immune decline, the mechanisms governing thymic adipose involution and its impact on thymocyte development warrant further investigation.

## Data Availability

The datasets presented in this study can be found in online repositories. The names of the repository/repositories and accession number(s) can be found in the article/supplementary material.
